# Small Open Reading Frames, How to Find Them and Determine Their Function

**DOI:** 10.3389/fgene.2021.796060

**Published:** 2022-01-28

**Authors:** Preeti Madhav Kute, Omar Soukarieh, Håkon Tjeldnes, David-Alexandre Trégouët, Eivind Valen

**Affiliations:** ^1^ Computational Biology Unit, Department of Informatics, University of Bergen, Bergen, Norway; ^2^ Sars International Centre for Marine Molecular Biology, University of Bergen, Bergen, Norway; ^3^ Department of Molecular Epidemiology Of Vascular and Brain Disorders, INSERM, BPH, U1219, University of Bordeaux, Bordeaux, France

**Keywords:** sORFs, ribosome profiling, mass spectrometry, computational tools, SEPs

## Abstract

Advances in genomics and molecular biology have revealed an abundance of small open reading frames (sORFs) across all types of transcripts. While these sORFs are often assumed to be non-functional, many have been implicated in physiological functions and a significant number of sORFs have been described in human diseases. Thus, sORFs may represent a hidden repository of functional elements that could serve as therapeutic targets. Unlike protein-coding genes, it is not necessarily the encoded peptide of an sORF that enacts its function, sometimes simply the act of translating an sORF might have a regulatory role. Indeed, the most studied sORFs are located in the 5′UTRs of coding transcripts and can have a regulatory impact on the translation of the downstream protein-coding sequence. However, sORFs have also been abundantly identified in non-coding RNAs including lncRNAs, circular RNAs and ribosomal RNAs suggesting that sORFs may be diverse in function. Of the many different experimental methods used to discover sORFs, the most commonly used are ribosome profiling and mass spectrometry. These can confirm interactions between transcripts and ribosomes and the production of a peptide, respectively. Extensions to ribosome profiling, which also capture scanning ribosomes, have further made it possible to see how sORFs impact the translation initiation of mRNAs. While high-throughput techniques have made the identification of sORFs less difficult, defining their function, if any, is typically more challenging. Together, the abundance and potential function of many of these sORFs argues for the necessity of including sORFs in gene annotations and systematically characterizing these to understand their potential functional roles. In this review, we will focus on the high-throughput methods used in the detection and characterization of sORFs and discuss techniques for validation and functional characterization.

## Introduction

An open reading frame (ORF) is defined as a start codon followed by a downstream in-frame stop codon. ORFs occur randomly and abundantly across the whole genome. Of these, only a fraction make their way into transcripts and only some of these end up being translated. Eukaryotic messenger RNAs predominantly have a single main ORF that make up its protein-coding sequence (CDS). The CDS is typically the longest ORF in the mRNA, but many other shorter ORFs are also often present in the transcript, some with the potential to be translated. Genome-wide studies have revealed the existence of many of these small ORFs (sORFs) in a wide variety of transcripts, including presumed non-coding transcripts, and that several of these are in fact translated.

**FIGURE 1:**
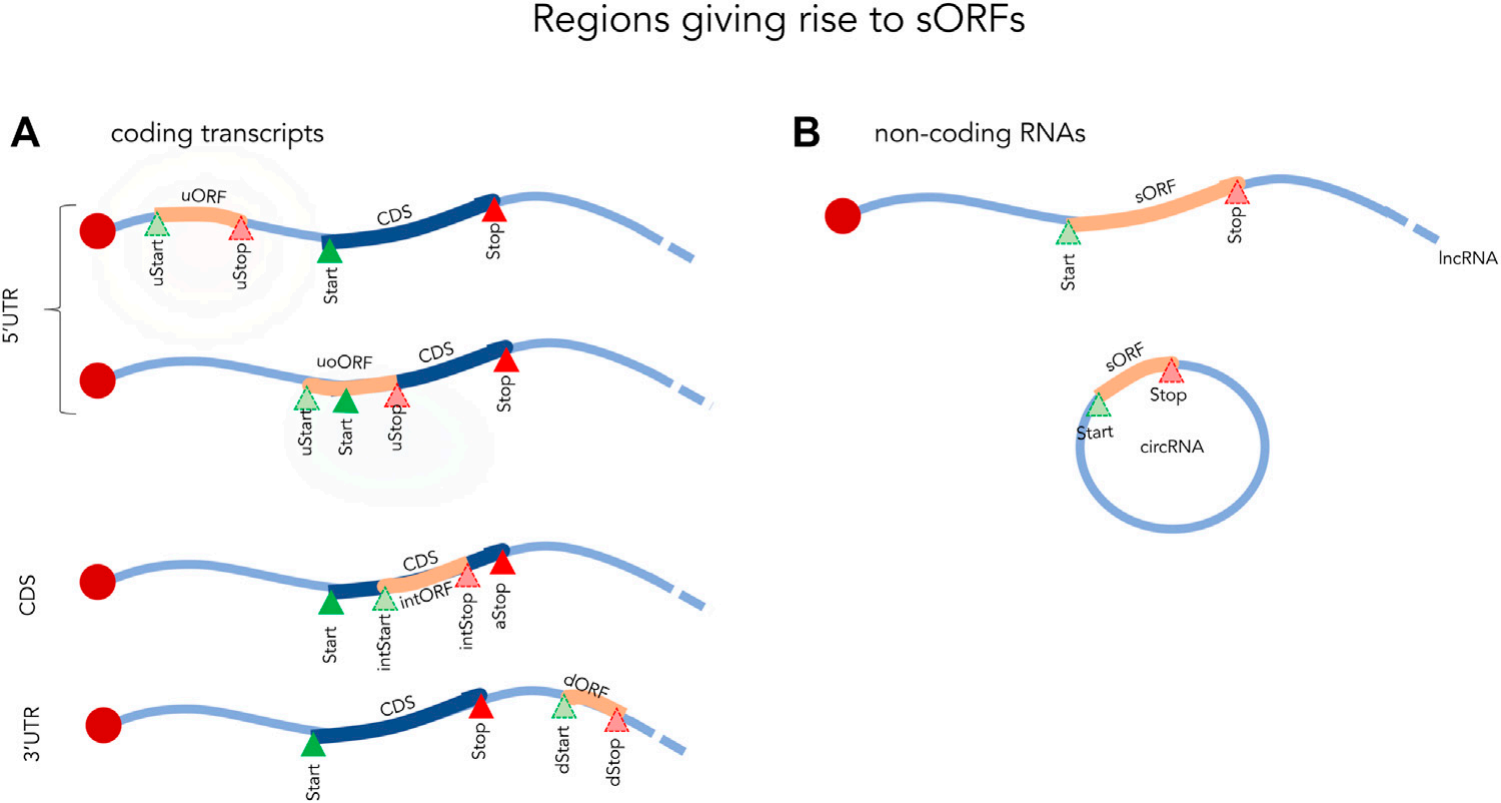
Examples of small ORFs in coding **(A)** and non-coding **(B)** transcripts. Start and Stop indicate the initiation and termination sites of the coding sequence (CDS). uORF, upstream open reading frame fully located in the 5′UTR; uStart, upstream start site; uStop, upstream stop site; uoORF, upstream overlapping open reading frame; intStart, internal start site; intORF, internal open reading frame; intStop, internal stop site; dStart, downstream Start site; dStop, downstream stop site; sORF, small open reading frame; lncRNA, long non-coding RNA; circRNA, circular RNA.

The most common definition of an sORF is simply an ORF of less than 100 amino acids (aa). These sORFs can be located within coding transcripts (5′UTR, CDS or 3′UTR) or even within non-coding RNAs such as long noncoding RNAs (lncRNAs), circular RNAs, and mitochondrial RNAs (Figure 1) (Orr et al., 2020). While some sORFs initiate with the canonical start codon (AUG), a significant number also initiate at near-cognate codons, differing by one nucleotide from AUG (Kearse and Wilusz, 2017). Of these, CUG, GUG, UUG and ACG appear to be the most frequent non-canonical translation initiation site in eukaryotes (Ivanov et al., 2011; Kearse and Wilusz, 2017; Cao and Slavoff, 2020). Translation termination sites typically use the conventional stop codons (UAA, UGA, and UAG), but studies have shown that sORFs can occasionally make use of unconventional termination (Cridge et al., 2018). Due to their small size and high abundance in most genomes (millions of sORF in eukaryotic genomes as reviewed in Couso and Patraquim, 2017), sORFs are often excluded from annotations in high-throughput analyses (Couso and Patraquim, 2017). In gene annotation pipelines, length cut-offs have traditionally been in common use (e.g., 100 aa) and anything below this threshold is typically considered to be non-functional.

Although it is challenging to characterize sORFs and to determine their potential functional role, several studies have now demonstrated the importance of sORFs in different cellular mechanisms (Zacharias et al., 2012; Zhang et al., 2018; Qin et al., 2018; Zheng et al., 2019b) and in the regulation of CDS translation (Calvo et al., 2009; van Heesch et al., 2019). While many of these sORFs function through their interaction with the ribosome and the resulting regulatory effect this enacts, some sORFs can also encode functional peptides. Several studies have identified sORF encoded peptides (SEP) demonstrating that many sORFs can indeed produce a peptide product. For instance, Ma et al. (2014) discovered 195 new SEPs in K562 human cells with only 29% starting with an AUG, the remaining having non-canonical start codons. Further examples of SEPs and their function is discussed in the last section of this review. Briefly, one can divide sORFs into different categories depending on their characteristics and the available evidence: 1) non-translated sORFs or those with no evidence of translation, simply defined from the genomic sequence (Young et al., 2015) 2) sORFs that are translated, possibly resulting in SEPs (van Heesch et al., 2019; Loughran et al., 2020) 3) sORFs and/or SEPs with a known function (Zacharias et al., 2012; Zhang et al., 2018; Qin et al., 2018; Zheng et al., 2019b; Cloutier et al., 2020). Taken together, this reflects a diversity of sORFs in both healthy and disease conditions and argues for a need to characterize them. The identification of sORFs and the determination of their translational status and functional role are of prime importance and their discovery is likely to reveal many new molecular players involved in regulatory mechanisms. In the following section, we will discuss the characteristics of sORFs and their location in the transcriptome.

## Small ORFs Are Abundant in 5′UTRs

The most highly studied sORFs are those found in the 5′UTRs of coding transcripts. These are referred to as upstream ORFs (upORF) as they are located completely or partially upstream of the main coding sequence (CDS). Depending on the location of the stop codon one can further distinguish upORFs into those that are (Figure 1): 1) completely upstream (uORF), where the sORF terminate before the CDS and 2) upstream overlapping (uoORF), where the sORF starts upstream, but extends out-of-frame into the CDS. Approximately, half of the human coding transcripts naturally contain upORFs (Calvo et al., 2009; Ye et al., 2015) and it is now evident that many of these affect the expression of the main protein (Calvo et al., 2009).

When ribosomes encounter upORF, several outcomes are possible (Hinnebusch et al., 2016; Silva et al., 2019). Most commonly, upORFs inhibit translation of the canonical protein by preventing some or all scanning ribosomes from reaching the CDS. This can be accomplished through numerous mechanisms such as ribosome dissociation (Grant and Hinnebusch, 1994), ribosome stalling (Law et al., 2001), RNA degradation through nonsense-mediated decay (Mendell et al., 2004), induction of ribosomes stalling and/or dissociation by the upORF peptide (Young et al., 2015) or by extending into the CDS and thereby preventing initiation (Lu et al., 2004). upORFs do not, however, always affect the translation of the canonical CDS. If the initiation context of the upORF is not optimal, the scanning ribosomes can potentially ignore the start codon of the upORF and continue to the CDS in a process known as “leaky scanning” (Palam et al., 2011). In the uORF case, with a stop codon upstream of the CDS, some ribosomes can also resume their scanning after translating the uORF and reinitiate at the CDS (Pöyry et al., 2004; Young et al., 2015). Which fate the ribosomes choose to depend on the features of the upORF and the transcript (Table 1), including the distance between the 5′cap and the upORF (Chappell et al., 2006), the strength of the upORF initiation sequence (Giess et al., 2020), and the strength of the upORF termination sequence (Giess et al., 2020; Wagner et al., 2020), the length of the upORF, the number of upORFs in the 5′UTR, and stable secondary structures located in the transcript (Wethmar, 2014). For instance, it has been shown that many transcripts with longer 5′UTRs are associated with a significant decrease of the main protein levels due to the presence of a high number of upORFs in the 5′UTR (Araujo et al., 2012; Giess et al., 2020).

**TABLE 1:** Transcript features defining the regulatory role of upORF. upORF, upstream open reading frame; uORF, fully upstream ORF; uoORF, overlapping upORF.

Feature	Comment(s)	Reference(s)
Secondary structures	Hairpin structures can function as inhibitors of translation initiation	Kozak, (1989)
The Kozak consensus sequence	Initially, the optimal Kozak sequence to initiate the translation was defined by a purine (R) at position -3 and a G in position +4 surrounding the translation initiation site (GCCRCCAUGG). However, recent Ribo-seq studies have shown that the optimal Kozak sequence could be different from the initially defined one, as shown in zebrafish by Giess et al., 2020	(Kozak, 1987; Giess et al., 2020)
Positioning of upORFs within the 5′UTR	overlapping upORFs are more often associated with repression of the main protein levels than non-overlapping upORF	Calvo et al. (2009)
Number of upORF	More upORF generally leads to more translational repression	Johnstone et al. (2016)
Length of upORF	Longer upORF is correlated with greater translational repression	Rajkowitsch et al. (2004)
Termination context of the upORF	The nucleotide context surrounding the uORF stop codon can affect translation reinitiation	(Hinnebusch et al., 2016; Giess et al., 2020; Wagner et al., 2020)

Recent studies have described the translation of small upORFs as a common event that can be initiated at both AUG and non-AUG codons (Slavoff et al., 2013; Rodriguez et al., 2019). Using a spectral coherence algorithm (SPECtre), Rodriguez and collaborators found that 4,954 upORFs are translated across 31% of all neuroblastoma transcripts, predominantly by using non-canonical start codons (Rodriguez et al., 2019). The resulting peptides can act as *cis*- regulating factors on the translation of the CDS. As an example, the SEP translated from an upORF in the 5′UTR of *GADD34* represses the translation of the CDS through induced ribosomal release mediated by a conserved 3 amino acid sequence at the C-terminal of this peptide (Young et al., 2015).

## sORFs Outside of 5′UTRs

Apart from upORFs, short out-of-frame ORFs located within the CDS (intORF, for internal out-of-frame ORF) and downstream ORF (dORF) located within the 3′UTR have also been identified in human coding transcripts (Couso and Patraquim, 2017; Wu et al., 2020). While these appear to be less abundant, they should not be ignored as some have been shown to have a regulatory function (Couso and Patraquim, 2017; Wu et al., 2020). For example, a dORF was recently shown to act as a translation enhancer of the CDS (Wu et al., 2020). By using a reporter assay in human cells, the authors showed a decrease in the expression of the CDS when inhibiting the translation of the dORF through mutating its start codon. This demonstrated a link between the translation of the dORF and an enhancing effect on the translation of the CDS.

Even though non-coding RNAs (ncRNAs) are defined by their lack of protein-coding potential, it is becoming increasingly clear that many of these contain sORFs that are recognized by ribosomes and result in the generation of SEPs (Ruiz-Orera et al., 2014). Such sORFs have now been found in most classes of ncRNAs, including lncRNA, circular RNAs, and ribosomal RNAs (Pang et al., 2018). While the evidence for translation for many of these is quite clear, we still do not know to what extent these sORFs are functional. Across ncRNAs, sORFs seem to be more frequent in lncRNA than in other non-coding RNA (Couso and Patraquim, 2017), likely due to their, on average, longer length. Encoded peptides from ncRNAs and their biological functions have been recently summarized in the review of Zheng et al. (2019a).

**FIGURE 2:**
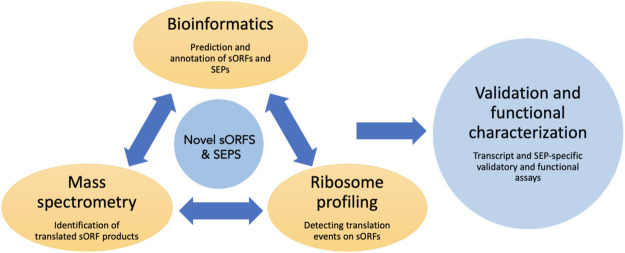
Overview of the commonly used techniques to identify and characterize sORFs and their encoded peptides. Novel sORFs and their products can be detected by the prediction algorithms using bioinformatic approaches, by generating peptide databases using improved mass spectrometry-based assays and by using ribosome profiling and related sequencing techniques to obtain translationally active transcripts. The predicted SEPs can be validated by various assays such as reporter-based overexpression, epitope tagging etc. Loss of function assays could be done to assess the cellular function of these SEPs.

In the following sections, we will discuss the various experimental and computational methods used to detect and identify sORFs, techniques for validation (summarized in Figure 2) and characterization and finally give some examples of functional sORFs.

## Discovering sORFs Through Ribosome Profiling

**FIGURE 3:**
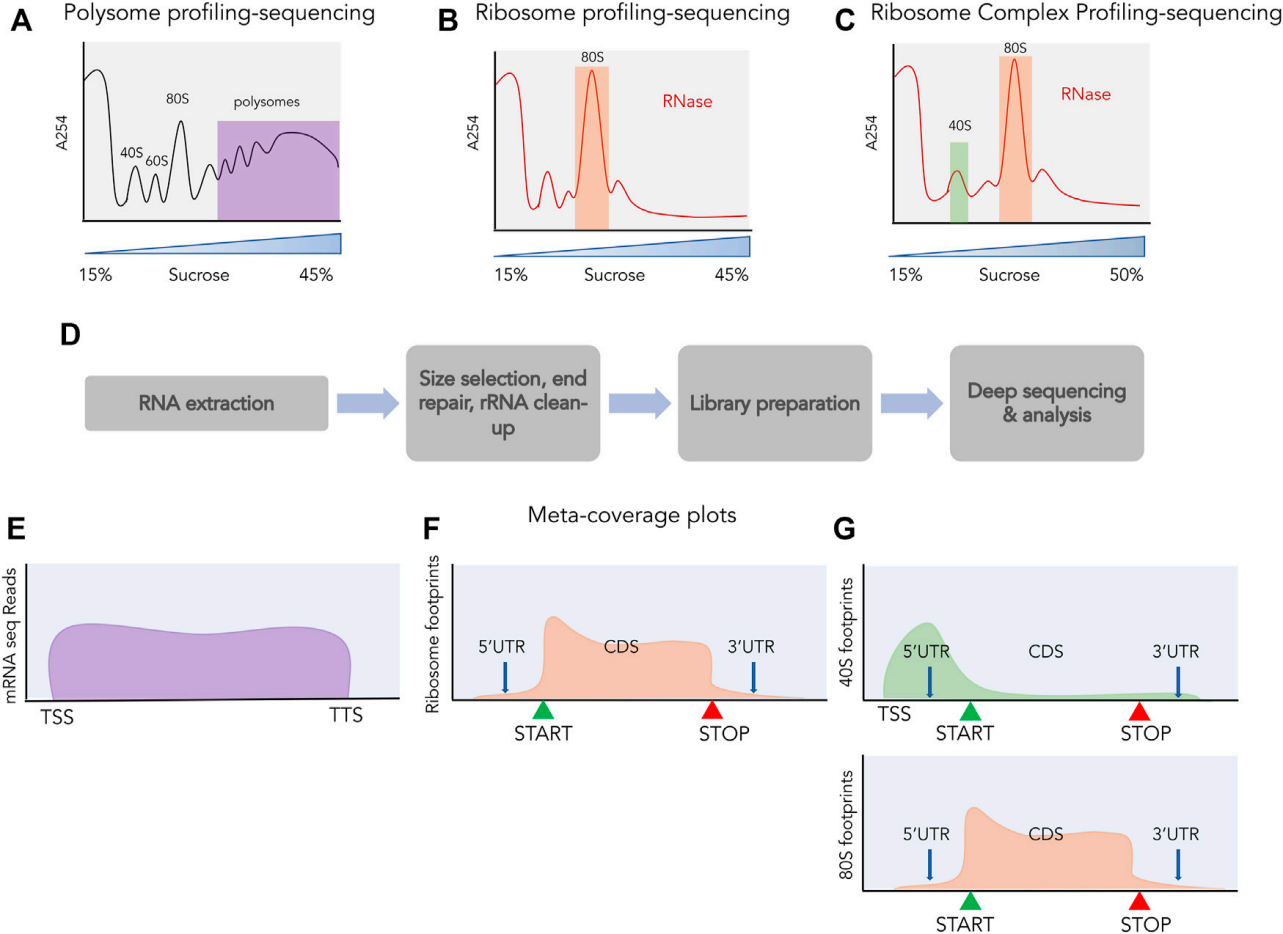
Profiling and sequencing of translating transcripts. A254 profiles shown before **(A)** and after digestion with ribonucleases **(B,C)**. The fractions used for further processing are highlighted, polysomes in purple, 80S in orange and 40S in green. **(D)** The process of library preparation for next generation sequencing. Size selection of ∼30 nt is done for ribosome profiling and ribosome complex profiling sequencing and libraries are prepared from the size selected small RNAs, whereas for polysome profiling, libraries are prepared from total RNA. Meta-coverage shown for reads obtained from polysome profiling sequencing **(E)**, for ribosome profiling **(F)** and for ribosome complex profiling [**(G)** top: 40S, bottom: 80S].

Although RNA-sequencing and mass spectrometric analysis quantify the abundance of RNA and proteins respectively, they do not provide information about the translational process itself. Among the many techniques used to monitor protein synthesis directly (Chekulaeva and Landthaler, 2016; Dermit et al., 2017), polysome profiling has played a key role in studying the perturbation of translation at the global level (Chassé et al., 2017). Although it is possible to combine transcriptomics and polysome profiling to perform global mRNA sequencing of polysomal fractions, this provides information only about the relative levels of transcripts (Figures 3A,E) across polysomes, not the positional information.

Ribosome footprinting is a classic biochemical assay where cellular mRNAs are digested with nucleases to selectively degrade RNA not protected by interaction with ribosomes and retain RNA fragments bound by ribosomes (Takanami et al., 1965; Steitz, 1969; Wolin and Walter, 1988). These ribosome-protected fragments (RPFs) are approximately 30 nucleotides in length and are obtained after nuclease digestion from the 80S fraction (Figure 3B). These RPFs can be converted into small DNA libraries which are then subjected to deep sequencing (Figure 3D). This technique of sequencing RPFs is called ribosome profiling or ribo-seq (Ingolia et al., 2009) and the reads obtained from such sequencing can be aligned back to the transcriptome providing a landscape of ribosomal occupancy across the whole transcriptome (Figure 3F). These reads provide both positional information such as where does translation take place and quantitative information such as how much is this region occupied by ribosomes. By normalizing these reads to the RNA abundance of the transcript they occupy, the translation efficiency of transcripts can also be estimated. Besides being an unprecedented tool for monitoring global translation, ribosome profiling has also shed light on the pervasive translation occurring outside of the annotated proteins. In the following sections, we will highlight studies that attempted to discover novel sORFs across the transcriptome using ribosome profiling and discuss extensions to this assay.

### Insights From Ribosome Profiling Studies on the Discovery of Novel sORFs

In the pioneering work introducing ribosome profiling, Ingolia et al. (2009) identified 1,048 putative upORF candidates initiating with the canonical AUG start codon, of which 153 candidates showed evidence of translation in *S. cerevisiae* (yeast). By further probing the 5′UTRs for non-AUG codons with strong initiation context and 28 nucleotide oligomer footprint alignment downstream of the initiation context, the authors found an additional 143 upORFs with evidence of translation. These included genes previously shown to harbor uORFs such as tRNA synthetases *GRS1* and *ALA1* in yeast (Ingolia et al., 2009). With the help of ribosome profiling, the authors were also able to decipher the role of the four known upORFs of *GCN4* in regulating the translation of the CDS on nutrient availability. While the upORFs are translated, the translation of the CDS of the *GCN4* transcript is inhibited during the log phase of growth, but during starvation, the upORFs are bypassed by the ribosomes and the CDS gets translated. Such detailed information about the choice of ORF translated in a single transcript is a good example of the many insights that can be uncovered using ribosome profiling.

Ribosome profiling has not only been used to visualize the distribution of ribosomes on coding transcripts, but also on non-coding transcripts. In an early study, Chew et al. (2013) produced ribosome profiling from early developmental stages in zebrafish and showed that the ribosome profiles of many lncRNAs were engaged with translating ribosomes. This work implied that many ORFs in lncRNAs are translated and could potentially either be novel genes or play a role in the localization/stability of the transcripts. A follow-up study showed that one of the annotated lncRNAs contained an ORF coding for a 58 aa long conserved peptide (Pauli et al., 2014) called Toddler. The translation product of this ORF was validated by mass spectrometric analysis and expression through GFP fusion constructs and functionally characterized revealing an important developmental regulator. Another study from the Pauli group using the same ribosome profiling dataset discovered yet another novel conserved peptide in a presumed non-coding transcript (Herberg et al., 2018). This peptide, called Bouncer, was shown to play a crucial role in preserving species-specific fertilization. The discovery of multiple uncharacterized novel genes encoded by sORFs from a single dataset illustrated the power of ribosome profiling in detecting novel translated regions. Later studies expanded on these discoveries and also made use of compounds to obtain specific subsets of ribosomes (Ingolia et al., 2011; Lee et al., 2012; Gao et al., 2015).

### Initiation Blockers Combined With Ribosome Profiling Identify Translation Initiation Sites

The most significant challenge when searching for novel sORFs is their size. Where canonical proteins often span thousands of nucleotides and can contain many RPFs, the RPFs mapping to sORFs are often scarce. Combined with noise from RNA structure and other RNA binding proteins which may generate protected fragments, this paucity can lead to difficulty in detecting high confidence sORFs. The majority of 5′UTRs also often contain many putative overlapping ORFs in all three reading frames making it difficult to assign an RPF to a specific upORF in order to determine which upORF, if any, are translated. Also, in cases where a single upORF have more than one potential start codon, the available RPFs are not always sufficient to determine which of these start codons is in fact the correct one or indeed if more than one is used.

To increase accuracy in determining initiation sites, and thereby gain more confidence in which upORFs are used, several studies have made use of initiation blockers that halt ribosomes at start codons and accumulate RPFs at these sites. This approach is especially helpful for sORF detection, where it is difficult to get good RPF coverage across the ORF due to their diminutive length. As one of the first high-throughput studies in the mammalian system, Ingolia et al. (2011) used mouse embryonic stem cells (mESCs) to perform ribosome profiling in the presence of harringtonine. Harringtonine binds to the free 60S subunit and blocks the formation of the bond between the initiator t-RNA to the A-site but allows the elongation to occur (Fresno et al., 1977). With the help of support vector machine (SVM)-based machine learning on a set of annotated genes, the authors identified features of RPFs that distinguished start codons from other sites. When applying this model to around ∼5,000 mouse transcripts, the model predicted that 65% of transcripts possessed more than one initiation site with 16% having four or more start sites. The authors also reported the presence of initiation sites at ORFs in most long intergenic non-coding RNAs (lincRNAs) and at upORFs in ∼68% of the 5,000 protein-coding annotated transcripts. Like previously observed in yeast (Ingolia et al., 2009), many of these sites were near-cognate and the authors further commented that since several non-AUG initiation events are resistant to harringtonine this could be an underestimate. Further analysis of the harringtonine-treated dataset showed that annotated start codons in protein-coding genes and randomly chosen harringtonine-predicted start codons in classical noncoding RNAs could not be distinguished (Guttman et al., 2013) suggesting that initiation blockers can be an important tool in identifying sORF start codons.

Other studies have refined this approach. Lee et al., 2012 attempted a more comprehensive mapping methodology by also using the initiation blocker lactimidomycin and comparing the translation initiation sites identified from this with those from harringtonine in HEK293 cells. Unlike harringtonine, lactimidomycin blocks the empty E-site of the 60S effectively blocking 80S elongation (Schneider-Poetsch et al., 2010). This technique was named global translation initiation (GTI)- sequencing (Lee et al., 2012) and was used to detect upstream initiation site in 54% of the transcripts studied, including that of the uORFs present in the gene *ATF4* (Lee et al., 2012)*,* a prominent model gene for uORF regulation (Lu et al., 2004)*.* By comparing the RPF patterns around annotated start codons in lactimidomycin and harringtonine, the authors concluded that harringtonine treatment led to the accumulation of ribosomes downstream of the start codon and therefore did not accurately predict the initiation site. While an improvement on the protocol exclusively using harringtonine, GTI-seq did not perform optimally in all cases. In perturbation experiments with serum starvation, it was found that GTI-seq obtained poor correlation (r = 0.069) between the RPFs over the lactimidomycin-identified start codons and the overall ribosome occupancy in the coding region (CDS). To overcome this shortcoming and obtain a better quantification of start codon use, lactimidomycin was combined with puromycin treatment which depletes elongating ribosomes (Fritsch et al., 2012). This new technique was named quantitative translation initiation (QTI) sequencing (Gao et al., 2015) and was applied to study the translational response to serum starvation in both HEK293 and mouse embryonic fibroblasts (MEFs) showing a marked improvement on the correlation obtained by GTI-seq (r = 0.375).

Other research based on the use of translation inhibitors have explored the side-effects of these inhibitors (Gerashchenko and Gladyshev, 2014; Kearse et al., 2019; Eisenberg et al., 2020; Enam et al., 2020). Thus, the use of these inhibitors requires careful optimization in terms of concentration, time of incubation and the system in consideration. Overall, however, the information obtained through translation initiation site mapping combined with RPFs obtained from ribosome profiling could be a valuable tool in mapping novel sORFs.

### Profiling of Small Ribosomal Subunits can Reveal sORF-Mediated Regulation

While ribosome profiling, both in its regular form and in the presence of initiation blockers provide information about the 80S elongating ribosomes, it only provides limited insight into the steps preceding or following translation elongation. Specifically, it does not capture intermediate ribosome complexes such as the pre-initiation complex, terminating and recycling ribosomal complexes. As upORFs can regulate the translation of CDS by inhibiting the ribosomes on their path to the start codon, obtaining global profiling of the scanning pre-initiation complex could provide in-depth analysis into the mechanism of translation regulation by upORFs.

A method for global profiling of scanning small subunits (SSUs) was developed by Archer et al., in 2016 and applied in yeast (Archer et al., 2016). By modifying ribosome profiling and separating the SSU from the 80S ribosomes post RNase1 digestion using sucrose density gradient, the authors were the first to map the SSU-bound mRNA footprints in a transcriptome-wide manner. This methodology was termed as translation complex profiling (TCP)- seq and provided global mapping of scanning complexes. These SSU footprints were abundant in the 5′UTRs, enriched around the start codons of transcripts and absent in the 3′UTRs. The original TCP-seq protocol only considered SSUs that were scanning on mRNAs that also contained an 80S elongating ribosome. A later study aiming to capture all SSUs, instead used the whole fraction of SSU without prior separation of free *vs*. 80S-bound in a method they called ribosome complex profiling (RCP-seq) (Giess et al., 2020) (Figure 3C). This study addressed the role of upORFs in regulating protein synthesis by measuring how many SSUs were “consumed” by the upORFs on their way to the CDS (Figure 3G). On an average, the SSU footprints density declined minimally across 5′UTRs except in the presence of an upORF, which also showed a concomitant increase of 80S footprints. The SSU loss was the highest for the upORFs starting with an AUG codon, but the study also demonstrated that the type of stop codon present in the upORF impacts the ability to reinitiate at the downstream CDS. For upORFs that contained a TGA stop codon, the least efficient, a higher rate of downstream translation could be observed and a lower rate of scanning indicating extended translation products. Together, this demonstrated that transcriptome-wide profiling of both 80S and SSUs can provide useful insights into sORF regulation and function.

### Selective Ribosome Profiling can Decipher the Function of Individual Factors

Recent studies have adapted TCP-seq to target selected initiation factors in a technique called selective-TCP-seq (sel-TCP-seq) (Bohlen et al., 2020; Wagner et al., 2020). By immunoprecipitation with antibodies against several of the translation initiation factors (eIF2S1, eIF3A, eIF3B, eIF4E, and eIF4G1) from the SSU and 80S fractions, the authors attempted to decipher the role of these factors in the scanning and initiation mechanism of the SSU and their role in re-initiation of translation for the CDS. Bohlen et al. (2020) showed that similar to the SSU at the start codon of the CDS, the SSU on the start codons of the translated upORFs have eIF3B, eIF4G1, and eIF4E, thus providing more insights into the translation of upORFs. Parallel studies trying to decipher the mechanism of upORF translation and translation re-initiation of CDS have revealed some of the molecular players participating in upORF translation such as the RNA helicase DDX3 and the re-initiation factors DENR and MCTS1 (Schleich et al., 2014; Schleich et al., 2017; Chen et al., 2018). Sendoel et al. (2017) showed that tumour initiation led to unconventional 5′UTR-mediated translation which is aided by the initiation factor eIF2A. Ribosome profiling studies from the malignant tissue showed the presence of footprints in the upORFs which coded for peptides. 13 of these upORF products were validated by mass spectrometry using the terminal amine isotopic labelling of substrates (TAILS) approach. Such knowledge of factors involved in upORF translation could be used to perform sel-TCP-seq for the factors of interest. Sel-TCP-seq could be used to pull down initiation factors such as eIF2A, DENR or MCTS1 that are involved in the translation of sORF to enumerate the sORFs present in human cells and to elucidate the mechanistic details of translation of such sORFs.

### Complementary Approaches to Ribosome Profiling

While ribosome profiling and its variants have provided a deeper understanding of the coding potential of the genome, these techniques are not without shortcomings. Ribosomal occupancy over a particular transcript does not necessarily imply true coding ability and the production of proteins. Ribosomes can be associated with the transcripts in a non-productive manner or ribosome association may have a regulatory role (Wilson and Masel, 2011; Johnstone et al., 2016). Additionally, RNA contaminants arising from structured non-coding RNAs or large ribonucleoprotein complexes co-precipitated with ribosomes may give false readouts of translation (Ingolia et al., 2014). To minimize noise from such interactions and discern true translation events, various approaches, both computational (discussed in a later section) and experimental, have been developed.

To address the issue of spurious, non-productive binding of ribosomes, several studies have made use of a more classical approach, polysome profiling (Aspden et al., 2014; Yang et al., 2018; Ye et al., 2021). In this approach, mRNAs are separated based on the number of ribosomes bound to them. Polysome-associated mRNAs, which are likely to be productively translated, can then be subjected to RNA-sequencing providing an estimate of their translation status (King and Gerber, 2016). Although mRNAs bound by multiple ribosomes and representing bonafide translation can be identified in this way, such datasets lack the positional information provided by ribosome profiling. Taking inspiration from ribosome profiling, Aspden et al. (2014) therefore, carried out nuclease digestion of the polysome fractions, in a technique they called Poly-Ribo-seq. This method was used to identify the translation of thousands of sORFs in *drosophila* S2 cells, which the authors categorized as long ORFs (∼80 amino acids) and dwarf ORFs (∼20 amino acids).

To deplete RNA contaminants originating from RNP complexes, Ingolia et al. (2014) introduced a variant of selective ribosome profiling using affinity purification of tagged ribosomes. Here the large subunit ribosomal protein L1 (formerly L10) was biotinylated *in vivo* in HEK 293 cells, and the ribosomes were purified by streptavidin pulldown. RPFs obtained from such affinity purification were deprived of the classical non-coding RNAs such as RNase P, which are known contaminants in conventional ribosome profiling datasets. Affinity purified profiling samples also lacked mitochondrial coding sequences since mitochondrial ribosomes lacked the biotin tag. Other studies have used enhanced GFP tagged ribosomal protein (RPL10a or RPL22) expressed in a cell-type specific manner that allows for monitoring cell type- or tissue-specific translation (Heiman et al., 2008), also known as translating ribosome affinity purification (TRAP-seq). By isolating ribosomes using anti-EGFP antibody-coated beads and using RNase digestion, the protocol was able to obtain cell- or tissue -type specific RPFs (Sapkota et al., 2019) which the authors termed as translating ribosome affinity purification-ribosome footprinting (TRAP-RF). Such additional tools and techniques can complement ribosome profiling and help identify novel sORFs and discern the true coding potential of non-coding transcripts.

## Validation of SEPs by Mass Spectrometry Technologies

While ribosome profiling-based approaches can reveal associations between ribosomes and RNA, another complementary approach to detecting translated sORFs is identifying the peptides resulting from their translation, the SEPs. While it is reasonable to expect a translated sORF to result in a SEP, due to the lack of conservation of sORFs, it is generally assumed that most of these SEPs are not functional. Furthermore, even if they are produced, many SEPs may be rapidly degraded. For individual SEPs, the use of overexpression constructs with reporter tags and fluorescence and epitope-based assays (discussed later) can be used to validate candidates. However, for large scale detection of SEPs, mass spectrometry (MS)-based techniques have been crucial. The recent developments in the MS field to accurately detect and validate SEPs have been well summarized in a recent review (Fabre et al., 2021). In the following section, we will discuss a few studies that have optimized MS to search for SEPs in human cells.

Conventional mass spectrometric based assays are not optimized to detect small peptides, as these small peptides may be degraded by peptidases, their levels may be masked by degraded products of other proteins, or they could remain undetected due to their low abundance. There are several approaches used to circumvent the problem of degradation of small peptides: alternative lysis methods such as boiling in hot water or trichloro acetic acid (TCA) and precipitation of proteins to denature endogenous peptidases (Slavoff et al., 2013; Wang et al., 2020). SEPs can then be enriched by size selection using ultrafiltration or by SDS PAGE separation for low molecular weight bands (Sapkota et al., 2019; Tharakan et al., 2020; Wang et al., 2020). Finally, instead of using trypsin to digest the peptides, other enzymes such as lys C were shown to increase the fraction of SEPs identified (Bartel et al., 2020). In addition, to better identify small peptides and avoid their degradation, peptidomics approaches inhibiting proteolysis that reduces the complexity of the proteome and using electrostatic repulsion hydrophilic interaction chromatography to separate peptides prior to HPLC- MS/MS has been used to identify novel SEPs (Slavoff et al., 2013).

In order to identify SEPs, mass spectrometry is often combined with RNA-seq techniques or ribosome-profiling to first annotate potential sORFs based on the nucleotide sequence and RPFs. For instance, several studies have utilized the published ribosome profiling datasets to search for SEPs and validate them by mass spectrometry. These studies have been summarized in Table 2. Interestingly, of the studies mentioned in the table, the micropeptide Nobody was successfully captured in both human and mouse systems (D’Lima et al., 2017; Budamgunta et al., 2018; Tharakan et al., 2020). Of note is a recent study in human iPSCs where Chen et al. (2020) combined ribosome profiling, the ORF-RATER algorithm, and MS-based proteomics to identify functional ORFs in different human cell types. Human leukocyte antigen class I (HLA-I) peptidomics approach was used to identify 240 novel peptides, some of which were validated to be lncRNA derived SEPs (ranging from size 55–124 aa) and upORF derived SEPs (ranging from size 15–70 aa). HLA-I based peptidomics has been successfully applied in other systems to identify SEPs, especially in tumour cells, thus implying that SEPs can be a source of antigens presented by T-cells (Bassani-Sternberg et al., 2015; Chong et al., 2020; Martinez et al., 2020). Oyama and others in their search for SEPs generated their own protein database through the 6-way translation of annotated RNA sequences to uncover non-annotated coding sequences in human cells. This approach of finding coding regions in the entirety of the RNA sequences led to the discovery of 54 SEPs out of which four were novel peptides in the human leukaemia K562 cell line (Oyama et al., 2004; Oyama et al., 2007). Additionally, by improving the SEP isolation and identification protocol, three studies from Table 2 have identified a total of 274 novel peptides (Slavoff et al., 2013; Ma et al., 2014; Ma et al., 2016) from human cell lines.

**TABLE 2:** Studies detecting SEPs through transcriptomic and/or mass spectrometry techniques.

Species	Technique	Number of SEPs discovered	Reference
Human	MS of HLA-I complexes	240	Chen et al. (2020)
Human	MS of HLA-I complexes and Ribo-seq	>500	Chong et al. (2020)
Human	MS of HLA-I complexes and Ribo-seq	320	Martinez et al. (2020)
Human	MS	1	D’Lima et al. (2017)
Human	MS and RNA-seq	>100	Ma et al. (2016)
Human	MS and RNA-seq	311	Ma et al. (2014)
Human	MS and RNA-seq	90	Slavoff et al. (2013)
Human	MS	197	Oyama et al. (2007)
Mouse	Ribo-seq and MS	1	Tharakan et al. (2020)
Mouse	MS	4	Budamgunta et al. (2018)
Zebrafish	Ribo-seq and MS	1	Pauli et al. (2014)
Zebrafish	Ribo-seq and MS	1	Herberg et al. (2018)

To optimize the identification of SEPs, the acquisition parameters can also be improved. Usually, data dependent acquisition (DDA) is the method of choice where tryptic peptides of top mass intensities in MS1 are chosen to be further fragmented in MS2. A study combined DDA MS analysis and optimized enrichment and extraction methods, to identify more than 100 SEPs in human cell lines (Ma et al., 2016). In contrast, data independent acquisition (DIA), in an advantageous manner selects a whole mass range for further fragmentation, increasing the chances to detect a single peptide. While this provides data with high coverage and precision this large dataset is highly convoluted and requires specialized data analysis (Bruderer et al., 2017; Fabre et al., 2017). Trapped ion mobility spectrometry (TIMS) using a time of flight (TOF) analyzer is another acquisition method, where the ionized molecules are separated in a gas phase. TIMS-TOF enhances peptide coverage and identification by resolving more ions, specifically isomers but with reduced chemical noise (Garabedian et al., 2018). Some of the features of both DIA and TIMS-TOF are promising in the quest of discovering low abundant small peptides.

Importantly, mass spectrometry can also be used to understand the interactome of SEPs, thereby opening avenues to the biological functions of SEPs. Further advances in the successful extraction of the small protein fractions and mass spectrometric detection undoubtedly will have a large impact on the field of protein function in general and in the context of human diseases.

## Computational Approaches Complementing Sequencing Techniques in the Search for sORFs

Classical gene prediction algorithms make use of numerous features common to the majority of genes, such as promoter sequences, polyadenylation signals, AUG start codons, codon bias and sequence conservation. Traditionally, however, these gene annotation pipelines have typically excluded ORFs shorter than 300 nucleotides since the lack of statistical power can make it hard to classify such short ORFs based purely on sequence (Frith et al., 2006). While pipelines for genome annotations are rapidly improving, in particular, due to the extensive sequencing of numerous genomes that can be used for comparative analyses, sORFs face many of the same problems that short genes have faced previously. sORF prediction is further complicated by the comparative lack of consensus features. For instance, many sORFs have non-AUG as start codons and have little to no sequence conservation (Ingolia et al., 2011; Lee et al., 2012), so searching for novel peptides is difficult using the standard features derived from protein-coding CDSs. Still, recent developments in computational approaches to predict sORFs have relied on gene-prediction methods such as detecting conservation of the sORF by comparison to other species, quantifying sORF codon bias and coding potential, and analyzing transcriptomic or proteomic datasets to identify sORFs that show evidence of translation. We will highlight some of the pipelines used to obtain predictions of sORFs and specifically focus on the algorithms going beyond sequence and using ribosome profiling to identify sORFs.

While most protein coding genes can easily be detected simply due to the unlikelihood of observing very long ORFs by random chance, functional sORFs are hidden in a genomes containing millions of similarly sized non-functional sORFs. Therefore, many methods exist to assess the conservation and coding potential of ORFs. An early tool was Coding Region Identification Tool Invoking Comparative Analysis (CRITICA), which analyzed synonymous and non-synonymous substitutions to predict proteins in the FANTOM collection of mouse cDNAs (Frith et al., 2006). Another computational tool called coding potential calculator (CPC) (Kong et al., 2007) defined six sequence features to distinguish non-coding from coding transcripts with the help of a support vector machine classifier. These features were calculated for the longest reading frame of a transcript and included determining its coverage (length relative to transcript length) and the extent of homologous protein sequences in other organisms. A later tool, PhyloCSF (Lin et al., 2011), featured a more direct conservation assessment quantifying the extent of synonymous to non-synonymous mutations based on sequence alignments. This method was shown to outperform other similar methods such as CSF (codon substitution frequencies) metric, PAML parameters, etc. and identified unknown SEPs. Finally, sORF finder (Hanada et al., 2010) is a bioinformatic package that uses nucleotide composition similarity to that of bonafide coding genes to identify sORFs. These potential sORFs are further tested for sequence conservation to assess their functional potential. While sequence-based predictors can be useful, additional accuracy can be achieved by combining these with information from ribosome profiling. For instance, one study used PhastCons, which can predict conserved elements from multiple sequence alignment (Siepel et al., 2005), together with ribosome profiling data available from mouse cell lines (Crappé et al., 2013) to gain more confidence in assigning a functional role to sORFs. However, more recent tools feature direct integration of ribosome profiling data in prediction pipelines. These ribosome profiling based tools can be broadly separated into two main groups: The first uses a variety of features of novel ORFs and compares these to known coding regions while the second is primarily oriented around finding periodicity in the ribosome profiling data caused by ribosome translocation.

One of the earliest examples of a ribosome profiling based ORF predictor is the translated ORF classifier (TOC) (Chew et al., 2013). This belongs to the first group of classifiers defining features based on patterns of ribosome footprints and using these to distinguish canonical annotated ORFs from those present in the UTRs. The authors defined two types of translation: coding and leader-like translation, where the latter was based on the translation patterns observed for uORFs. ORFs from transcripts were then classified into either of these categories or non-coding. While transcripts predicted to be coding resembled classical genes, leader-like transcripts were shown to frequently have more than one sORF. A similar observation was made by Guttman et al. (2013) based on their ribosome release score (RRS). This metric was based on the fact that ribosomes are typically released after translating a protein and was defined as the ratio of the total number of reads from the putative coding region to the number of reads from the putative 3′UTRs. Although some of the non-coding RNAs showed ribosomal occupancy at similar levels to protein-coding genes (Ingolia et al., 2011), they scored significantly lower than protein-coding genes on the RRS metric (median score ∼1 versus ∼112 for proteins). Other studies have argued along the same lines that many non-coding transcripts are associated with ribosomes in a non-productive manner and either do not undergo active translation or do not result in functional peptides (Wilson and Masel, 2011). In an attempt to increase accuracy from regions covered by RPFs, Ingolia et al. (2014) developed a metric to distinguish genuine 80S footprints from non-ribosomal sources of footprints based on the footprint size distribution. Fragment length organization similarity score (FLOSS) measures the degree of disparity between the length distribution of footprints obtained for an abundant transcript and the characteristic RPF size (26–34 nt). In yet another study, Bazzini et al. set out to estimate the coding potential of ORFs by utilizing the bias in read distribution introduced by the translocation of the ribosome and developed a metric to capture this (ORFscore). This was combined with a tool to detect conserved peptides: micro-peptide detection pipeline (micPDP) (Bazzini et al., 2014). ORFscore identified 303 novel protein-coding transcripts out of the 2,450 previously predicted ncRNAs. micPDP based on PhyloCSF identified 63 conserved zebrafish peptides with only 23 overlapping with those found by ORFscore.

The second group of ribosome profiling ORF classifiers uses spectral analysis of nucleotide periodicity to detect statistically significant regions with a 3 nucleotide periodic signal. Among these are SPECTre (Chun et al., 2016) RiboTaper (Calviello et al., 2016), ORFquant (Calviello et al., 2020), Ribotricer (Choudhary et al., 2020) and RiboNT (Song et al., 2021). RiboNT and Ribotricer both apply weighted codon scores to mitigate noise, Ribotricer, in addition, presented evidence for sustained recall when the size of ORF is decreased (dropping at < 20 codons). Although these state-of-the-art tools perform well on typical cases, none of them have been benchmarked on more complex cases such as highly overlapping sORFs and very short sORF, where periodicity is limited (Brar and Weissman, 2015). It is therefore unclear how well prediction tools work for these more complex cases. Complete annotation of sORFs therefore, remains a challenging task and as argued by an earlier review (Pauli et al., 2014) a combination of these ribosome profiling based predictors together with sequence-based metrics are likely to yield the most robust performances.

Beyond detection, more general analysis frameworks for ribosome profiling are implemented in the python package Plastid for exploratory data analysis (Dunn and Weissman, 2016). The Bioconductor package ORFik, uses ribosome profiling datasets to quantify ribosome elongation and RCP-seq and TCP-seq datasets to quantify ribosome scanning and initiation (Tjeldnes et al., 2021). For proteomic validation, tools such as PinStripe and PROTEOFORMER can be used to predict and validate sORFs at the proteomic level. (Gascoigne et al., 2012; Verbruggen et al., 2019). These proteomics tools can add a layer of verification but can be challenged with short ORFs since the probability of random hits increases. Table 3 categorizes these tools depending on the input dataset requirement (sequence, Ribo-seq and/or proteomic data) and summarizes the output of these tools. Finally, sORF databases such as sORFs.org and smPROT have compiled most of the studies to generate a repository of sORFs discovered in various model systems (Olexiouk et al., 2016; Olexiouk et al., 2018; Hao et al., 2018). This reflects the effort to study sORFs and the need to identify and characterize sORFs to discover the hidden/neglected parts of the human genome and their crucial role in gene regulation and diseases.

**TABLE 3:** Overview of the computational tools aiding in the prediction of sORFs.

Method	Features utilized	Input requirement	Output dataset	Reference and links
Sequence-based prediction tools				
CPC2	Nucleotide composition, sequence similarity	RNA-seq	Coding potential of especially lncRNAs	(Kang et al., 2017) and GitHub
micPDP	Codon conservation	RNA-seq	sORF detection from non-coding RNA	Bazzini et al. (2014)
PhyloCSF	Codon substitution	RNA-seq	Coding potential	(Lin et al., 2011) and GitHub
PhastCons	Nucleotide composition	Whole genome	Conserved elements, especially signatures outside a protein-coding region	(Siepel et al., 2005; Crappé et al., 2013) and GitHub
sORF finder	Nucleotide composition similarity	Any nucleotide sequence	sORFs	(Hanada et al., 2010) and Link
Ribosome profiling-based tools				
FLOSS	Ribosome fragment length	Ribo-seq	True ribosome footprints	Ingolia et al. (2014)
ORFscore	3-nt periodicity	Ribo-seq	Ribo-seq ORFs	Wu et al. (2020)
ORFquant	3-nt periodicity, transcript features such as exonic bins and splice junctions	Ribo-seq	Ribo-seq ORFs on multiple transcript isoforms	(Calviello et al., 2020) and GitHub
ORF-RATER	Read density over start and stop codons	Ribo-seq	Ribo-seq ORFs	(Fields et al., 2015) and GitHub
RiboTaper	3-nt periodicity	Ribo-seq, RNA-seq	Ribo-seq ORFs	(Calviello et al., 2016) and Link
RiboNT	3-nt periodicity (noise tolerant), codon usage	Ribo-seq	Ribo-seq ORFs	(Song et al., 2021) and GitHub
Ribotricer	3-nt periodicity	Ribo-seq	Ribo-seq ORFs, especially sORFs	(Choudhary et al., 2020) and GitHub
RRS	Read density drop after stop codon	Ribo-seq	Ribo-seq ORFs	Guttman et al. (2013)
SPECtre	3-nt periodicity	Ribo-seq	Ribo-seq ORFs	(Chun et al., 2016) and GitHub
TOC	Ribosome footprint patterns	Ribo-seq	Ribo-seq ORFs	Chew et al. (2013)
PROTEOFORMER	3-nt periodicity, Mass spec hits	Ribo-seq, Mass spec	Ribo-seq ORFs, MS ORFs	(Verbruggen et al., 2019) and GitHub

## Validation and Functional Characterization of sORFs

Beyond high-throughput detection, many techniques can be used at the individual gene level to validate sORFs, their interaction with ribosomes and their potential translation into SEPs. These techniques include toeprinting in its classical and fluorescent versions (Koš et al., 2002; Egorova et al., 2019), epitope tagging (Aspden et al., 2014), *in vitro* translation (Raney et al., 2000), and proteomic peptide phage display (Garrido-Urbani et al., 2016). While these validate the translation of an ORF, additional experiments are necessary to determine the potential function of an identified sORF. Most of these approaches are similar to determining the function of ordinary genes but with the added complexity that sORFs may share their transcript with other ORFs and often do not exert their function through its peptide product. Also, even though an upORF may exerts its function through its peptide product, perturbing it in order to functionally characterizing it may have side effects that affect the stability or translation of the main CDS, confounding the process of uncovering the upORFs function.

Other common approaches for functional assays include cellular localization assays and CRISPR-mediated knockdown. A more unconventional approach, however, was used in a recent study that employed antisense oligonucleotides (ASOs) against upORFs to upregulate the expression of the CDS (Liang et al., 2016). While the mechanism is not precisely understood, upORF-targeting ASOs have been used to restore levels of the main protein by modulating the efficiency of ribosome initiation at the upORF (Liang et al., 2016; Liang et al., 2017). Thus, ASOs are a functional tool that can be used to assess the effect of a given upORF on the expression of the CDS.

### Implication of sORFs and Their Encoded Peptides in Humans: Relevant Examples

Translation of sORFs into SEPs has been demonstrated in many coding and non-coding transcripts but their functions are not systematically determined. Many examples of human translated sORFs from upORFs in coding genes or intergenic sORF with well-identified functions have been reviewed (Andrews and Rothnagel, 2014). More recently, another review summarized the main functional SEPs, and the methods used for their identification (Yeasmin et al., 2018). These techniques are mainly based on the identification of SEP-protein interactions. For instance, to reveal interactions between the C11orf98 micropeptide from C11orf98 gene and proteins, proteomic analysis, immunoblotting and immunoprecipitation experiments have been performed on cells transfected with constructs containing the micropeptide with a tag (APEX, ascorbate peroxidase 2) associated with biotin-phenol labelling (Chu et al., 2017). Thus, the authors discovered the interaction of the micropeptide with nucleolar proteins nucleophosmin and nucleolin. Moreover, the authors confirmed the association of the 69 aa long modulator of retroviral infection (MRI) protein with Ku70 and Ku80, suggesting its implication in DNA repair (Chu et al., 2017). That shows the utility of defining SEP-associated proteins as a powerful hypothesis-generating approach.

Another example is the 46 aa long SEP, Myoregulin (MNL) encoded from a presumed lncRNA and shown to be expressed in skeletal muscle and involved in the regulation of Ca2+ handling by inhibiting the pump activity of SERCA, a membrane pump that controls muscle relaxation by regulating Ca2+ uptake into the sarcoplasmic reticulum (SR) (Anderson et al., 2015). Very recently, Koh and collaborators identified short ORF-encoded histone binding protein (SEHBP) as a transcriptional regulator (Koh et al., 2021). In their study, the authors developed an experimental assay identifying partners of SEP in cells by introducing a photo-crosslinking non-canonical amino acid into SEP transgenes and using enhanced affinity purification mass spectrometry-based mapping strategy. This method allowed the authors to identify the interaction between SEHBP and chromatin-associated proteins. Further, transient overexpression of SEHBP-eGFP in human cells, followed by RNA-seq showed a significant modulation of the transcript levels suggesting a role of SEHBP in the transcription regulation.

In a recent study, Sun et al. (2021), identified an onco-micropeptide APPLE (90 aa) encoded by the non-coding RNA *ASH1L-AS1* using a combinatorial approach of ribosome profiling, mass spectrometry and RNA-seq analyses. The authors showed that APPLE is overexpressed in subtypes of acute myeloid leukaemia and led to a poor prognosis. Functional assays showed that APPLE exhibited a pro-cancer role both in *in vitro* and *in vivo* models of acute myeloid leukaemia. Using sub-cellular fractionation, APPLE was identified to be in the endoplasmic reticulum and by interacting with poly-A-binding protein C (PABPC1), enhanced the translation and synthesis of certain oncoproteins. By regulating a specific pro-cancer translation program, APPLE may be one of several undiscovered SEPs, playing a crucial role in cancer biology.

In some cases, SEP could act as antigens recognized by immune cells and that could be used as targets in therapy. For instance, Charpentier and collaborators have described the generation of three small peptides from three sORF located within the lncRNA *Meloe* (Charpentier et al., 2016). These small peptides have been described as antigens implicated in melanoma. Starck and his collaborators have shown that uORFs of the BiP transcript act as (HLA)-presented epitopes recognized by human T cells (Starck et al., 2016). Also, small peptides deriving from mitochondrial DNA (Humanin and MutS-C), called mitochondria-derived peptides (MDPs), have been described to have a protective role in cardiovascular diseases (Yang et al., 2019). Most of the characterized human SEPs and their physiological and functional roles have been reviewed recently (Wright et al., 2021).

## Conclusion

In this review, we have summarized computational and experimental techniques that can be used for the identification and characterization of small ORFs and their encoded SEPs. Most of the well performing methods use a combination of sequence information paired with data from ribosome profiling. Based on these predictions, validation assays such as mass spectrometry and epitope tagged expression analysis can provide the concluding evidence of the presence of sORF encoded peptides. Indeed, the application of these techniques in different species has led to the identification of several sORFs and SEPs. Beyond identification, high-throughput techniques such as TCP-seq and RCP-seq and selective ribosome profiling can be used to probe the function of the sORFs at the genome-wide level in different disease models and even patient samples. We have further given examples of sORFs showing their importance in a wide range of contexts. By now, several studies have demonstrated the diversity of sORF function and their role in normal and disease contexts, arguing that sORFs are abundant in many genomes and significant efforts should be put towards their annotation and characterization.
